# Questionnaires to Measure Process and Structure of Quality Indicators for Pediatric Nursing

**DOI:** 10.1097/pq9.0000000000000381

**Published:** 2020-12-28

**Authors:** Maria Forsner, Evalotte Mörelius, Lena Hanberger

**Affiliations:** From the * Department of Nursing, Umeå University, Umeå, Sweden;; † Department of Biosciences and Nutrition, Karolinska Institutet, Stockholm, Sweden;; ‡ School of Nursing and Midwifery, Edith Cowan University, and Perth Children’s Hospital, Perth, WA, Australia; and; § Department of Health, Medicine and Caring Sciences, Division of Nursing, Linköping University, Linköping, Sweden

## Abstract

The quality of nursing care has a significant impact on the outcomes of care. The specific needs of children requiring hospital care make it essential to monitor and compare data not only on the medically oriented outcome measure but also on nursing care, structure, and process, requiring perspectives from registered nurses (RNs) and nurse managers (NMs). Thus, this project aimed to evaluate the structure and process of nursing quality indicators in pediatric hospital care with questionnaires distributed to RN and NM.

**Methods:**

We developed separate questionnaires for NMs and RNs to assess the process and structure of the quality indicators of breastfeeding, management of pain, venous access, medication management, and provision of a child-oriented environment. Nine NMs and 113 RNs from 9 pediatric wards answered the questionnaires.

**Result:**

Local guidelines were available for 3 out of the 5 quality indicators: pain management, venous access, and medication management. RNs reported varying levels of adherence to pain management (62%), and venous access management (72%). Satisfaction with the conditions for safe medication management was 90%. Approximately, two-thirds (67%) of RN reported sufficient knowledge regarding the impact of the child-oriented environment and less than half (44%) regarding how to support breastfeeding.

**Conclusion:**

Structure and process is a prerequisite for quality of care outcomes. This study discloses areas for quality improvement and offers instruments to compare structure and process in pediatric nursing care to discuss with consumers, managers, staff, and other stakeholders.

## BACKGROUND

Nursing care has a significant impact on patient safety and the outcome of care.^1^ However, quality outcome data on nursing activities mostly refers to adult care^2^^,^ and pediatric quality of care measures are mostly medically oriented.^3,4^ This situation jeopardizes the quality of nursing care to children and conflicts with the child’s best interest.^5^ Against this background, the Swedish Association of Pediatric Nurses has drawn attention to the absence of pediatric nursing care indicators, stating that this makes the quality of children’s care invisible to consumers and stakeholders and undermines comparison within and between hospitals.

Indicators of quality of health care require scientific plausibility, relevance, and consistent interpretability. They ought to be unique, measurable, and valid.^6^ From the perspective of children, communication, professional competence, safety, professional appearance, and virtues such as patience, honesty, and kindness are essential factors for quality in nursing care,^7^ and technical skills, and the on-time delivery of medications.^8^ Moreover, children want to take an active part in decisions about their own care.^7–9^ From a parental perspective, parents likewise appreciate being heard, nursing professionalism, and technical skills. Furthermore, parents emphasize the importance of support and information.^10^ From a nurse perspective, family presence, safe medication administration, pain management,^11^ and peripheral venous catheter management are key quality indicators.^3^

On behalf of the Swedish Association of Pediatric Nurses, a working group including nurses experienced in different pediatric nursing fields, such as general pediatrics, diabetes care, neonatal intensive care, nursing management, and quality improvement, was mandated to identify quality indicators for nursing in pediatric hospital care. The group agreed to focus on: breastfeeding, pain management, safe venous access, safe medication management, and providing a child-oriented environment (Table 1). These indicators are within a registered nurse’s responsibility, and they are generic and valid across different pediatric specialties.

**Table 1.** Defined Indicators and Targets for Nursing in Child Hospital Care

Quality Indicator	Target
Breastfeeding	Breastfeeding is established and sustained in children treated in hospitals
Management of pain	Wherever possible, children are relieved from pain and are given to understand that their experience of pain is taken seriously, and their pain is appropriately alleviated
Safe venous access	Venous access is safe and without complications, and handled with respect for the child’s needs
Safe medication management	The right medication at the right dose is administered correctly to the right patient at the right time
Providing a child-oriented environment	The hospital environment is tailored to the child’s and parents’ needs and supports safe care

Furthermore, the group was guided by the Donabedian Quality-of-Care Framework, of which structure, process, and outcomes are essential components.^6^ *The structure* provides conditions for good health. The organization, staffing, facilities, equipment, expertise, and procedures, that is, providing guidelines are some aspects falling under “structure.” *The process* assesses healthcare operations performed, including all activities during the meeting with the patient and family, such as sampling, examinations, and treatments according to guidelines. *Outcomes* include improvement or change in the patient’s health attributed to the efforts of care.^6^

The Baby-Friendly Hospital Initiative and strategies such as the Integrated Management of Childhood Illness highlight the importance of breastfeeding.^12^ *Pain* can significantly impact the individual child. It is associated with morbidity, affects survival, and the quality of life.^13^ *Venous access* has been designated as challenging in pediatric care and is a common source of complications.^14^ The fact that children are at exceptionally high risk of *medication errors*, which are more hazardous to children than to adult patients, is also well known.^15^ Furthermore, the hospital environment is crucial to meet children’s specific needs,^16^ and children themselves emphasize its importance.^17^

Healthcare should be grounded in scientific knowledge, proven experience, and the patient’s perception of the required care. Additionally, health services should provide equal quality of care for the whole population. Therefore, it is of great importance to survey patient experiences and monitor and compare outcome data on the healthcare provided, highlighting areas in need of improvement. However, according to the Donabedian Quality-of-Care Framework,^6^ measuring process and structure are just as important as measuring outcomes. Furthermore, Batalden and Davidoff^18^ emphasize measuring quality improvements from different perspectives. Thus, with a focus on structure and process, both registered nurses (RNs) and nurse managers (NMs) perspectives were of interest. This study aimed to evaluate the structure and process of nursing quality indicators in pediatric hospital care with questionnaires distributed to registered nurses and nurse managers.

## METHODS

### Questionnaires

Two questionnaires, 1 for NMs and 1 for RNs, were used to assess the structure and process of the quality indicators: breastfeeding, pain management, safe venous access, safe medication management, and providing a child-oriented environment. Two authors (L.H. and E.M.) developed the questionnaires in collaboration with an expert group of 8 RN specialists in pediatric nursing with clinical and research experience.

The questionnaires were initially tested on 1 NM and 5 RNs from 1 pediatric clinic, resulting in some linguistic adjustments and removal of overlapping questions. Items already addressed in deviation management systems or measured by the healthcare organization (eg, concerning adherence to hygiene routines) were also excluded. After that, M.F. performed cognitive interviews at 1 Neonatal Intensive Care Unit (NICU) and 1 pediatric ward in another hospital. Two NMs and 7 RNs, 5 of whom were specialists in pediatric nursing, participated and were asked to think aloud when answering the questionnaire. The interviews were tape-recorded and analyzed item by item using the Respond Problem Matrix, coding for lexical, temporal, logical, omission/exclusion, and/or computational problems.^19^ One omission problem was disclosed, which highlighted a need to define “mother-child co-care.”

The final version of the questionnaire for NMs included 27 questions about breastfeeding (n = 11), pain management (n = 5), safe venous access (n = 3), safe medication management (n = 3), and providing a child-oriented environment (n = 5). Answer alternatives were yes/partly/no/not applicable.

The final version of the questionnaire for RNs included 9 questions about breastfeeding (n = 1), management of pain (n = 4), safe venous access (n = 2), safe medication management (n = 1), and providing a child-oriented environment (n = 1). Answer alternatives were yes/always/partly/no/do not know/not applicable.

### Participants

Three different hospitals, including 9 family-centered pediatric wards in the south of Sweden, participated in the survey. The wards included 2 NICUs caring for newborns admitted directly from the delivery room, 3 outpatient clinics, and 4 inpatient care wards for children 0–18 years. We invited all NMs and RNs from the 9 wards to complete the questionnaires. In Sweden, RNs are responsible for breastfeeding consultation, nonpharmacological pain management, insertion of venous catheters, medication administration, and providing a child-oriented environment (process). The NMs are responsible for providing conditions for optimal care (structure).

### Procedure

During the spring of 2013, we sent an email to the nurse responsible for quality improvement at each hospital with a request to forward the invitation to NMs and RNs at the clinic. Following the Helsinki Declaration, the email also included information about the purpose of the study, an assurance that participation was voluntary, completely anonymous, and that the respondents gave consent by submitting the questionnaire. The respondents got access to the electronic questionnaire through a link in the email.

## RESULTS

All the 9 NMs representing each unit and 113 RNs completed the questionnaire. The RNs worked in outpatient care (n = 37) and inpatient care (n = 76).

### Quality Indicator: Breastfeeding

Among the 6 NMs representing inpatient wards, 4 reported that they had guidelines for breastfeeding support, 5 reported that they include breastfeeding in all memos about nutrition. Four reported that they had available updated parent information about breastfeeding at the ward. Six NM reported that mothers had access to a private, comfortable chair appropriate for breastfeeding and were allowed to stay with their child around the clock if they breastfed their child. Two NMs reported the availability of a structured method to perform breastfeeding observations. Four NMs reported that they documented goals and actions to promote and sustain breastfeeding in the care plan (Table 2). One NM commented: “We perform breastfeeding observations when needed, but structured methods to perform breastfeeding observations combined with structured documentation could be improved.” All 6 NMs of inpatient wards reported that they document breastfeeding at discharge and that the staff’s knowledge about breastfeeding was evaluated regularly (Table 2).

**Table 2.** Questions Measuring Structure and Responses from the NMs

Questionnaire Item	Yes, n	Partly, n	No, n	Not Applicable, n	No Answer, n
On your ward: Are guidelines for breastfeeding support available?	4	0	2	0	3
Is breastfeeding considered in all memos about nutrition?	5	0	0	0	4
Is a structured method for performing breastfeeding observations available?	2	0	4	0	3
Do the care plans include goals and actions to promote and sustain breastfeeding?	4	0	0	0	5
Is updated parent information about breastfeeding available?	4	0	2	0	3
Do mothers have access to a private, comfortable chair appropriate for breastfeeding?	6	0	0	1	2
Can mothers stay with their hospitalized child round the clock to promote breastfeeding?	6	0	0	0	3
Is breastfeeding documented regularly?	6	0	0	0	3
Is the staff’s knowledge about breastfeeding evaluated regularly?	6	0	0	0	3
Are there validated pain assessment tools available?	9	0	0	0	0
Are there guidelines for pain management available?	8	0	0	0	1
Is there a member of staff with special responsibility for pain management?	1	0	7	0	1
Is pain specifically documented in the patient records?	5	0	1	0	3
Are there routines for pain assessment?	6	0	2	0	1
Are there guidelines for venous access technique available?	9	0	0	0	0
Is updated parent information about venous access available?	4	0	5	0	0
Is written age-appropriate information for children about venous access available?	6	0	2	0	1
Are local guidelines for medication management available?	9	0	0	0	0
Is there an undisturbed place for the preparation of medications?	5	4	0	0	0
Do nurses regularly perform knowledge tests about medication calculation?	1	0	7	0	1
Is there a possibility for parents to sleep close to their hospitalized child during the night?	5	0	0	3	1
Is child impact analysis performed and considered when a change is made in the hospital unit environment?	4	0	4	0	1
Are patients, parents, and youth advisory boards represented in the planning of a potential change?	4	0	4	0	1

The NMs’ responses to the question, “Of the children who were breastfed at the time of admission (NICU not included), how large a proportion (%) were breastfed at discharge?” were qualitative rather than quantitative.

Among the RNs, 44.2% felt that they had sufficient knowledge about breastfeeding to support and advise parents (Table 3).

**Table 3.** Questions Measuring Process and Responses from the RNs

QUESTIONNAIRE ITEM	Yes, Always, n (%)	Partly/ Occasionally, n (%)	No, n (%)	Do Not Know, n (%)	Not Applicable, n (%)	No Answer, n (%)
Do you feel you have sufficient knowledge about breastfeeding to support and advice parents?	50 (44.2)	30 (26.5)	17 (15)	0	16 (14.2)	0
Do you follow the local guidelines for pain management?	70 (62)	18 (16)	0	5 (4.4)	8 (7)	12 (10.6)
Do you prevent expected pain related to procedures?	76 (67.2)	25 (22.1)	0	0	1 (0.9)	11 (9.7)
Do you evaluate the effect of pain prevention?	54 (47.8)	42 (37.1)	1 (0.9)	0	3 (2.6)	13 (11.5)
During the last 5 y, have you attended any training on pain management in children?	33 (29.2)	0	68 (60.2)	0	0	12 (10.6)
Are you satisfied that the requirements for safe and secure medication management are met in your ward?	46 (40.7)	56 (49.6)	10 (8.8)	0	1 (0.9)	0
Do you follow the guidelines for venous access?	82 (72.6)	16 (14.2)	0	5 (4.4)	6 (5.3)	4 (3.5)
Do you have sufficient competence in venous access technique?	90 (79.6)	14 (12.4)	4 (3.5)	0	2 (1.8)	3 (2.6)
Do you have sufficient knowledge about the impact of a child- oriented environment?	76 (67.2)	32 (28.3)	5 (4.4)	0	0	0

### Quality Indicator: Management of Pain

Guidelines for pain management and validated pain assessment tools were available in all the wards (Table 2). Of the RNs, 62% reported complete adherence to the guidelines, while 16% reported that they followed the guidelines occasionally (Table 3). Some of the reasons for nonadherence were: “cannot find the guidelines”; ”as far as I know, we do not have guidelines”; “sometimes there is an acute situation, and there’s no time”; and “sometimes the child/adolescent wants to proceed without pain prevention.”

It was more common for RNs to prevent expected pain (67.2%) than to evaluate the results of pain prevention (47.8%) (Table 3). Some reasons given for refraining from preventing expected pain were: “it is an acute situation”; “sometimes the physician acts before I have time”; “it is difficult to prevent pain during capillary blood sampling”; “lack of time”; and “sometimes the child/youth refuses to take pain medication.” Among the reasons given for failing to evaluate pain prevention were: “it is not always possible to get a reliable evaluation because of the difficulties to communicate with the patient/parents, for instance, if the family does not speak Swedish” and “I try to remember, but I need to prioritize, and assessment isn’t a top priority.” Also, RN reported evaluating pain management but not always using pain assessment tools, and one RN commented: “I do evaluate pain, but I’m bad at documenting the results.”

Of the RNs, 29.2% had attended and passed a course or training involving children and pain management during the last 5 years (Table 3).

### Quality Indicator: Safe Venous Access

Information about venous access for children of different ages was available on 6 wards. Information for parents about venous access was available in fewer than half of the wards (Table 2). Two comments from the NMs were: “the children are too small, but it is a good idea to have information for parents” and “it’s not applicable in our department, but maybe we should have information for siblings.”

Among the RNs, 72.6% reported complete adherence to venous access guidelines, whereas 14.2% reported that they occasionally followed the guidelines (Table 3). One RN reflected: “the purpose is to follow the guidelines, but sometimes you just have to think out of the box and find individualized solutions.” Of the RNs, 79.6% reported enough competence in the venous access technique (Table 3). Two reasons RNs gave for not having enough competence were: “there are so many kinds of peripheral venous catheters” and “there are always new products on the market, which makes it difficult to stay up to date.”

### Quality Indicator: Safe Medication Management

According to the NMs, all wards had local instructions about routines for safe medication management. Approximately, half of the wards provided a place where the RNs could prepare medications without distraction. Only 1 ward used knowledge tests on medication calculation for RNs (Table 2).

Altogether 90.3% of the RNs were completely or partly satisfied with the possibilities to perform safe and secure medication management (Table 3). However, comments from RNs pointed in another direction: “there are too many medications, which makes it easy to mix up different strengths”; “it’s difficult to find what you need in the medication room”; “you are often disturbed by colleagues or parents”; and “when the ward is busy the conditions for safe preparation of medications are bad, and they’re getting worse.”

### Quality Indicator: Providing a Child-oriented Environment

Five NMs reported that parents could stay close to their child at night, 1 did not respond, and 3 answered: “not applicable” because they worked in daycare units. Four NMs reported that a child impact analysis was regularly performed and considered when instituting changes in their department, including parents, children, and youth, giving their opinions on advisory boards. One NM implied that she did not know what a child impact analysis was.

Two of the questions posed to the NMs gave qualitative instead of quantitative responses, namely: “What proportion (%) of parents stay close to their child 24 hours a day at the NICU?” and “What proportion (%) of mothers who have recently given birth stay with their child at the NICU?” One NM commented on the first question: “We have enough room for 100% of the parents, but we have not measured if they stay.” On the second question, one NM stated: “We have not measured here either.”

Among the RNs, 67.2% reported sufficient knowledge about providing a child-oriented environment, 28.3% reported some knowledge, and 4.4% reported insufficient knowledge (Table 3).

## DISCUSSION

The questionnaires addressed the process and structure of the quality indicators of breastfeeding, management of pain, safe venous access, safe medication management, and provision of a child-oriented environment. Their importance for nursing care quality is well established.^3,7–11^ The reason why we focused on structure and process was that this gives essential information about why quality goals are achieved or not. Because NMs are responsible for the organization, facilities, equipment, procedures, staffing numbers, and competence, we approached them to measure the structure of nursing quality. To approach the process measures, we asked for nurses’ self-assessment of activities directed to the patient and family during direct nursing care.

All units had guidelines for breastfeeding, pain management, peripheral venous catheter technique, and medication management. In line with previous research,^20^ this survey showed insufficient adherence to guidelines for pain management. The fact that not all nurses reported following the pain management guidelines increased the risk of adverse pain experiences for the children. Slightly more but still, an insufficient number of RNs were reported to follow the venous access guidelines, which contrasts with previous results reporting a 70-100% adherence from different wards.^21^ As part of the Donabedian framework structure, the idea of guidelines is to give the standard for the care and reduce variations in the care provided and, therefore, help ensure the quality of care.

All units except the NICUs had age-appropriate information for children about venous access, but few units distributed written parental information. Interestingly, the survey seemed to inspire the recipients to introduce new materials for parents and siblings, an example of how the questionnaire might add to quality improvement. Another example is that 1 NM regularly gave nurses knowledge tests on medication calculation. Sharing such information might stimulate others to copy the idea.

The World Health Organization^22^ highlights the importance of breastfeeding knowledge among the staff at all units delivering care to children under 2 years of age. However, RNs reported insufficient knowledge about breastfeeding support. Moreover, missing responses from NM about the proportion of breastfeeding at admission and discharge were explained by the fact that they did not record these measures at that time. Breastfeeding knowledge is essential for quality pediatric care, and the lack of responses highlight the need for further quality improvement in this area.

The participating wards practiced family-centered care with single family-rooms, so parents could stay around the clock, access school, and play therapy. Still, there was a gap in the RNs’ knowledge about the impact of a child-oriented environment on children’s health, indicating a need for more attention to this area in both research and practice. A child-friendly, family-centered, colorful, and exciting, playful environment might positively influence the children’s emotions and cause less stress, anxiety, and fear, thus facilitating the hospital visit experience. However, as mentioned by Water et al,^23^ we should not underestimate the importance of respecting children’s rights to dignity, privacy, family support, and self-control.

### Limitations

The fact that some years have passed since the survey has to be considered. Because clinical nursing practice changes continuously, it is essential to implement measurements of the structure and process of pediatric nursing care quality. However, because Brenner et al^24^ confirmed these indicators, the questionnaires developed in 2013 could still be judged as relevant. House et al^4^ recognize the importance of implementing measures on structure and process besides outcome measures and adverse events to target areas for improvement. All the proposed indicators, besides breastfeeding, can be judged as relevant for children up to 18 years of age. However, because of all pediatric units participating in this study care for children from birth, breastfeeding was also considered relevant.

Other limitations to consider were (1) the use of self-rating questions without objective verification (such as observation studies) of the findings or the outcomes of care increases the risk for bias; (2) children and parents were not represented in the design; and (3) the response rate from RNs is unknown because we do not know how many RNs were working at the time of the survey. The number is unknown because the email distribution list also included casual staff, and staff on parental leave, sick leave, and annual leave. Furthermore, some questions to the NMs do not apply to outpatient care, which should be considered when distributed to NM working in outpatient care.

We recommend using a Likert scale to enable statistical calculations of comparisons between wards and hospitals for future work. Moreover, repeated or continuous measures of structure and process would enable an analysis of correlations with outcome data, adverse hospital events, and child/parent experiences.

## CONCLUSIONS

Structure and process is a prerequisite for quality of care outcomes. This study offers questionnaires to compare structure and process in pediatric nursing care as a basis for discussions with consumers, managers, staff, and other stakeholders. External factors may influence these indicators, but providing guidelines and policies offers better possibilities for safe care. In this study, local guidelines were available regarding pain assessment tools, pain management, peripheral venous catheter technique, and medication management at almost all wards. Still, there was a need for greater adherence to guidelines and increased knowledge regarding breastfeeding. Pinpointing gaps in care can be used to stimulate new research ideas. However, to fully evaluate the pediatric nursing care provided, there is a need for asking the children themselves and their parents.

## DISCLOSURE

The authors have no financial interest to declare in relation to the content of this article.

## ACKNOWLEDGEMENT

This study would not have been possible if the Swedish Association of Pediatric Nurses had not initiated the work. We would like to thank the remaining working group members Britt-Marie Ygge, Susanne Åkerström and Christina Sandström for their contribution. We would also like to thank the participants who took the time to contribute and answer the questionnaires in this study. The project was presented at the Swedish annual Pediatric Meeting (Barnveckan) in 2014 and awarded as the best nursing poster. The price sum was 4000 Swedish Crown (SEK).
